# Pelvic Symmetry Is Influenced by Asymmetrical Tonic Neck Reflex during Young Children’s Gait

**DOI:** 10.3390/ijerph17134759

**Published:** 2020-07-02

**Authors:** Ewa Gieysztor, Anna Pecuch, Mateusz Kowal, Wojciech Borowicz, Małgorzata Paprocka-Borowicz

**Affiliations:** 1 Physiotherapy Department, Faculty of Health Sciences, Wroclaw Medical University, 50-355 Wroclaw, Poland; anna.pecuch@student.umed.wroc.pl (A.P.); mateusz.kowal@umed.wroc.pl (M.K.); malgorzata.paprocka-borowicz@umed.wroc.pl (M.P.-B.); 2 Department of Nervous System Diseases, Faculty of Health Sciences, Wroclaw Medical University, 50-367 Wroclaw, Poland; wojciech.borowicz@student.umed.wroc.pl

**Keywords:** gait symmetry, pelvic symmetry, preschool children, neurodevelopment, asymmetrical tonic neck reflex

## Abstract

Gait is one of the examined functions in child development. It should be economical and symmetrical. One test increasingly used by physiotherapists and pediatricians is asymmetrical tonic neck reflex (ATNR). Physiologically, it is observed from in utero up to six postnatal months. This reaction is inhibited with the growing maturation of the central nervous system (CNS). In some children, when the natural process of development is incorrect, ATNR manifests later in life, when it is observed as an automatic response of muscle tension to head rotation. Analysis of pelvis symmetry in the gait of children with active ATNR is important for better understanding their specific movements. In the gait of children with persistent ATNR, some variations are observed. The aim of the study was to investigate the gait symmetry of preschool children and the influence of persistent ATNR. Fifty preschool children with a trace form of ATNR were examined. The distribution of the gait parameters was determined using a BTS G-SENSOR measurement instrument. ATNR negatively influences pelvic obliquity and pelvic rotation (*p* < 0.01). Younger children have a statistically higher symmetry index of pelvis obliquity in the examined group (*p* = 0.015). Boys obtain a higher result of symmetry in pelvic tilt than girls in the group (*p* = 0.027). ATNR affects walking symmetry in preschool children, thus evaluation of the reflex activity and then proper therapy is required to support proper development.

## 1. Introduction

Gait symmetry is one of the determinants of proper child development. Some asymmetries are normal for human movement, however some can be impacted for various reasons. For parents and pediatricians as well as physiotherapists, the wide spectrum of factors influencing child development should be known for proper movement analysis [1]. Persistent asymmetrical tonic neck reflex (ATNR) is one of the factors that can impact child development. It has been found very commonly in healthy preschool children. The most frequent type is the left side form of this reflex [2,3,4,5]. The activity of the reflex during the motion gives some additional muscle tension response, which involves the central nervous system (CNS) after reception of additional stimuli. The movement of the head, in people with active ATNR, induces movement or muscle contraction in the limbs and trunk. This is especially visible in closed-chain tasks. The head rotation provokes extension activity in the muscles of the limbs and trunk at the face side and flexion muscle activity at the occipital side of the body. The grade of the response depends on the degree of primitive reflex (PR) activity. The more vital the reflex, the more active the response, which is visible as increased muscle tone, even causing motion in the limbs and the trunk [3,4].

The most studied symptoms connected with ATNR activity refer to learning difficulties such as visual disturbances, poor stability of posture during sitting and writing, and large and small motor skill delays or inadequacies [4,6,7,8]. Difficulties in riding a bicycle are also observed in children with ATNR.

The impact of ATNR on gait symmetry has not yet been studied, as far as the authors could determine. Noticing the wide range of child developmental fields impacted by primitive reflexes (PRs), the authors investigated if the walking pattern is also regulated by and dependent on the degree of PR activity. Because gait is a complex motion, which involves the whole body, dependence on the reflex reaction through muscle tension may influence gait parameters [1,3,9,10]. Thus, the aim of the study was to find if there is an ATNR impact on the symmetry parameters of the gait. Moreover, we wanted to check if there is a difference between two types of ATNR testing (in closed- and open-chain) taking into consideration the correlation with pelvic symmetry index parameters.

## 2. Materials and Methods

The study was approved by the Wroclaw Medical University Ethical Committee and conducted in accordance with the Declaration of Helsinki. All the parents of the subjects were kept informed of the purpose and process of examination and subsequently gave their written consent prior to the study.

### 2.1. Participants

In the study, fifty children (30 girls and 20 boys) were examined. The mean age of the group was 5.5 (±0.5) years. The mean height of the participants was 100 cm (±10), and the mean weight was 17 kg (±3.4). The group had ATNR at different levels, from none to extreme activity, according to the Goddard scale [11]. Parents reported neither pathological lower limb abnormalities at the time of testing nor other musculoskeletal/neurological and/or cardiopulmonary conditions likely to influence walking level.

### 2.2. Measurement of Asymmetrical Tonic Neck Reflex

The examination of ATNR was conducted in two different ways. The first test was executed in quadruple position according to the Ayres recommendation, and the second in standing position, using the Schilder test [12]. The Ayres test is taken in closed-chain, and the Schilder test is performed in open-chain.

In the Ayres test, the child was asked to stay in quadruple position. The researcher turned his or her head to the left and then to the right side. After turning the child’s head, the researcher observed whether there was any change in upper limb position. The five-degree scale of reflex activity is: 0 for full integration to 4 for maximum activity. The child with ATNR shows occipital-side elbow banding from the mildest form, to elbow banding and shoulder banding with trunk rotation in the maximum form of activity.

In the Schilder test, the child was recommended to stand straight, feet close together, arms flexed to 90 degrees, hands facing down. The researcher turned the child’s head to both sides slowly. During the test, the arms follow the head if active ATNR is observed. Maximum points are given for turning the whole body while the head is changing position.

### 2.3. Pelvis Symmetry Gait Parameters Measurement

The acquisition of the pelvis symmetry gait parameters was performed using a BTS G-SENSOR measurement instrument (BTS Bioengineering Corp., Quincy, MA, USA). The device was equipped with a Triaxial Accelerometer 16bit/axis with multiple sensitivity (±2, ±4, ±8, ±16 g), a Triaxial Gyroscope 16bit/axis with multiple sensitivity (±250, ±500, ±1000, ±2000 °/s), as well as a Triaxial Magnetometer 13bit (±1200 uT). An inter-instrument correlation coefficient between 0.90 and 0.99 and an intra-instrument coefficient of variation of ≤2.5% proved the G-Sensor to be suitable for the assessment of physical activity [12,13,14].

The measurement of gait was conducted with the protocol for the walking test. Children were asked to walk in their natural way four times over a distance of five meters. Children were walking barefoot. The pelvis symmetry parameters that were analyzed were: pelvic tilt (S), pelvic obliquity (F), and pelvic rotation (T).

### 2.4. Statistics

Statistical analysis was performed using IBM SPSS Statistics version 25 (IBM Corp., Armonk, NY, USA). Arithmetic means and standard deviations were calculated for continuous variables. In order to determine the relationship between quantitative variables, Spearman’s rho correlation analysis was used. Mann–Whitney U test was used to compare two groups in terms of quantitative variables. The chi-square test of independence was used to compare groups in terms of nominal/categorical variables. The level α = 0.05 or α = 0.01 was used for comparisons.

## 3. Results

### 3.1. Profile of Asymmetrical Tonic Neck Reflex in Examined Group

**Figure 1:**
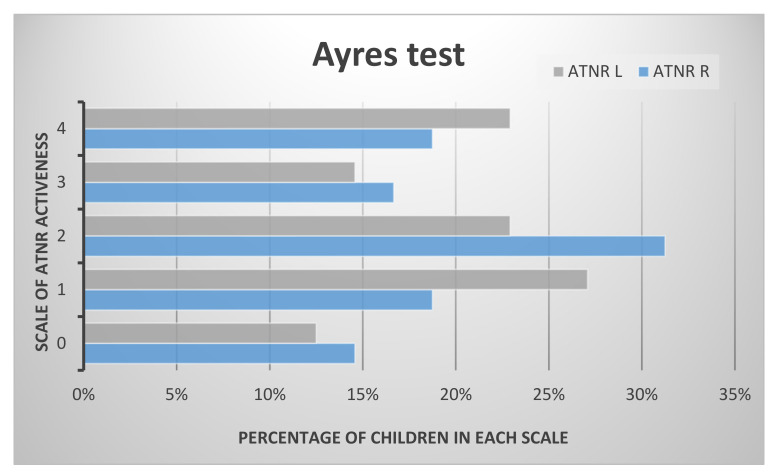
The results of asymmetrical tonic neck reflex (ATNR) in the examined group in the Ayres test.

**Figure 2:**
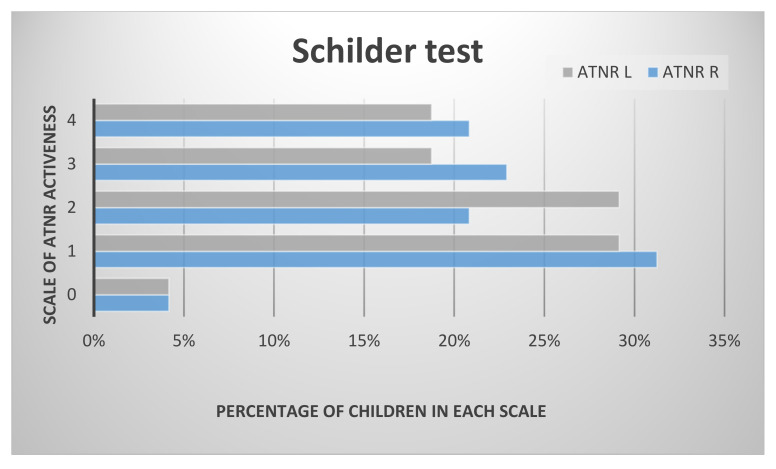
The results of ATNR in the examined group in the Schilder test.

Using the Ayres test to examine the activity of asymmetrical tonic neck reflex, the results show that nearly 15% of examined children had no activity of the ATNR. In the Schilder test it was under 5% of examined preschoolers. The biggest group of children in both tests had ATNR R pointed at 2 in Ayres and 1 in the Schilder test. The results indicate that about 20% of the preschool children show the maximum intensity of ATNR, in both tests. The results of asymmetrical tonic neck reflex tests in the examined group of preschoolers are shown in Figure 1 and Figure 2.

The correlation between the results of the Ayres test and the Schilder test was significant (*p* < 0.01). Moreover, the correlation between gender and ATNR measured in quadruple position was significant. Boys had higher results for ATNR than girls.

### 3.2. Pelvic Motion during Gait

The results of pelvic symmetry in means, maximum, minimum, and standard deviation of the examined children are shown in Table 1.

**Figure 3:**
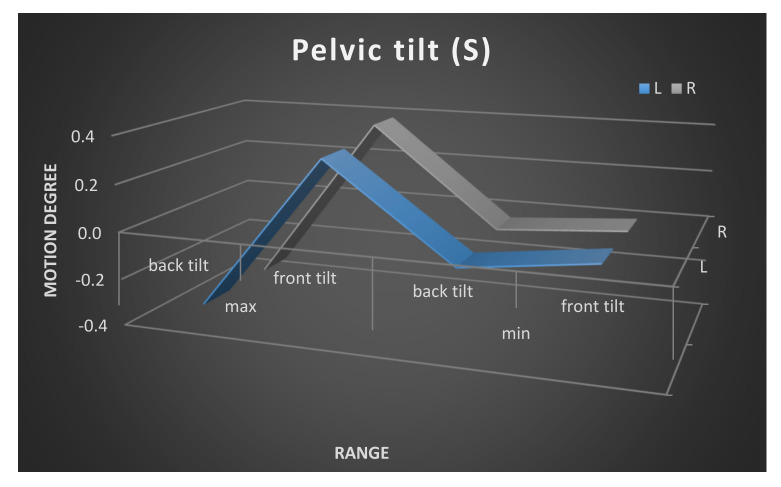
Pelvic tilt maximum and minimum range of movement. Left and right side comparison.

**Figure 4:**
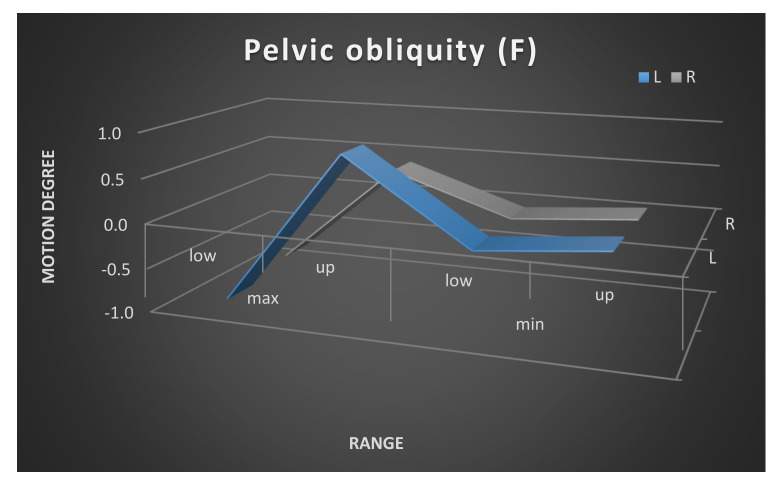
Pelvic obliquity maximum and minimum range of movement. Left and right side comparison.

**Figure 5:**
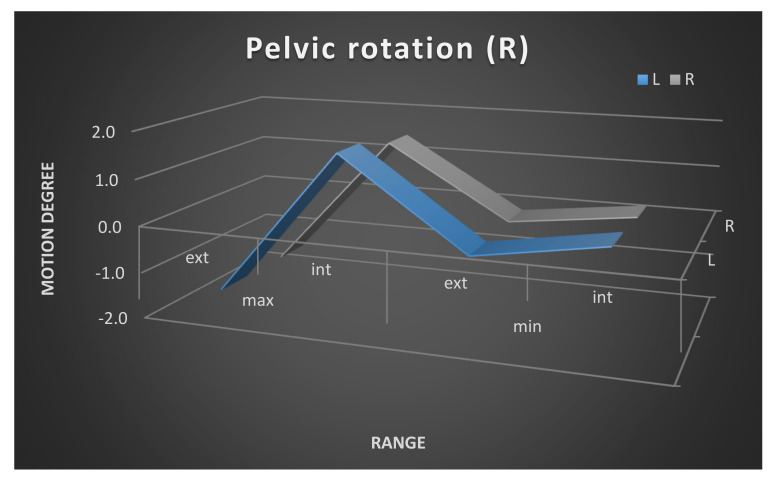
Pelvic rotation maximum and minimum range of movement. Left and right side comparison.

Moreover, the mean maximum and minimum range of the pelvic movement on the left and the right sides is shown in Figure 3, Figure 4 and Figure 5. The pelvic rotation had the highest mean range of movement and the least had pelvic tilt. The least symmetry in the mean range of two sides movement of the pelvis was observed in frontal plane. Clear difference is between maximum of high position of the pelvis in the left and the right side.

### 3.3. Correlation Between Asymmetrical Tonic Neck Reflex and Gait Parameters

Correlation analysis using Spearman’s rho coefficient was carried out to check the relationship between gait parameters and persistent asymmetrical tonic neck reflex. Table 2 contains the results of the analyses carried out. Statistically significant correlations are observed for ATNR and pelvic obliquity as well as pelvic rotation. For ATNR Left Side measured both in closed- and open-chain, the results are nearly statistically significant for pelvic rotation. ATNR R and ATNR L in all the other results are strongly and moderately negatively correlated with pelvic obliquity and rotation. The results show no correlation between ATNR and pelvic tilt.

### 3.4. Correlation between Age and Gait Parameters

To check whether age was related to gait parameters, a correlation analysis was performed taking into account Spearman’s rho coefficient. The analysis shows that the pelvis symmetry parameters did not correlate with age in the examined group.

**Figure 6:**
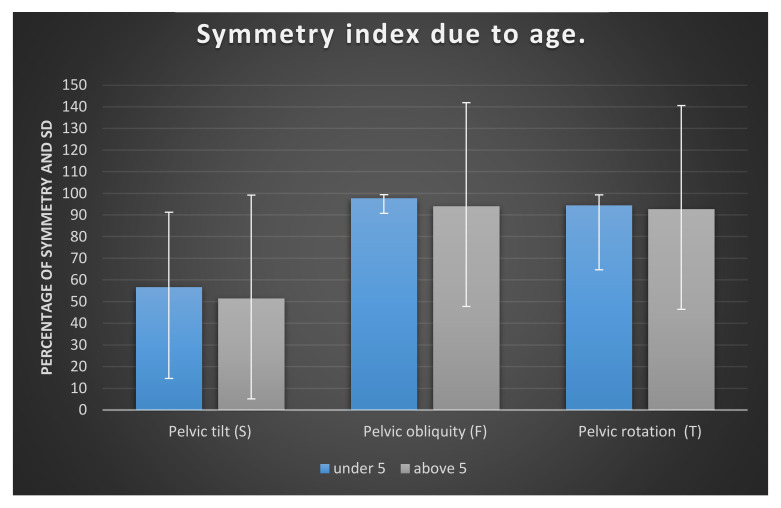
Symmetry index of pelvic tilt, pelvic obliquity, and pelvic rotation in examined children, shown as mean according to age.

Additionally, the respondents were divided into two age groups: children under 5 years of age (*n* = 23) and above 5 years of age (*n* = 27). The results for gait parameters in both groups were compared using the Mann–Whitney U test. Differences were observed between the two groups of younger and older children in the parameter of pelvic obliquity (PO). Children under 5 years old have a statistically higher symmetry index of PO in the examined group; Figure 6 and Table 3 displays the results.

### 3.5. Differences between Girls and Boys in Pelvis Symmetry Parameters

**Figure 7:**
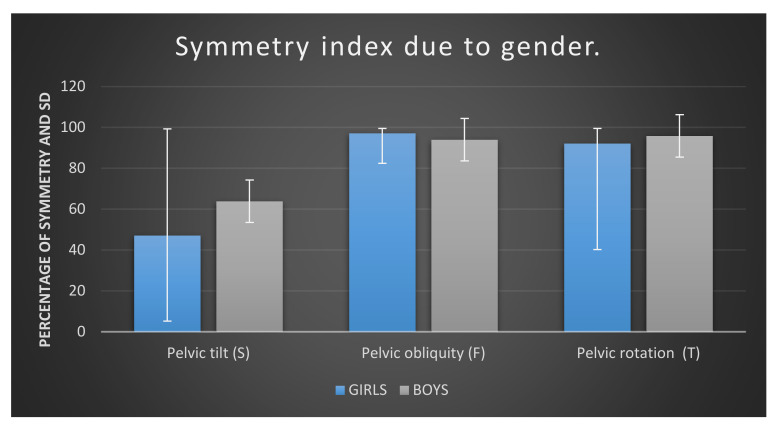
Symmetry index of pelvic tilt, pelvic obliquity, and pelvic rotation in examined children, shown as mean according to gender

To determine whether girls and boys differed in terms of gait parameters, an analysis was carried out using the Mann–Whitney U test. A graphic representation of the results for girls and boys is shown in Figure 7. The most different results in these two groups are observed in the sagittal plane. In addition, the standard deviations of the results are higher in girls. SD results in boys more closely correspond to the mean in all pelvic movements.

Detailed analysis of the results is presented in Table 4.

The analysis showed one significant difference between the girls and boys examined. This difference relates to symmetry in pelvic tilt (S). Boys obtain a significantly higher result for this parameter than tested girls.

## 4. Discussion

Persistent primitive reflexes (PPR) co-exist with disturbances in child motor and posture development [4,15]. Their activity indicates the level of neuromotor immaturity in children [6,16,17,18]. Some children with PPR are observed to move in a specific way during spontaneous play as well as during gait. They have no neurological or musculoskeletal orthopedic impairments, but their movement schema differs. The question is if their movement can be indicated just by muscle tension caused by reflex response? Is the specific way that children with primitive reflexes perform walking visible in the gait parameters?

The results of our research show that primitive reflexes, such as ATNR, if they persist, have a significant impact on symmetry parameters in pelvis kinematics during gait. It seems that gait is another field of child development which is impacted by ATNR. Previously, learning skills have been studied, as well as eye–hand coordination and motor skills [2,3,4,19,20,21,22,23]. The kind of measurement that we proposed was conducted for the first time, so there is no strict comparison with other research. The most common gait analysis is conducted in children with scoliosis or cerebral palsy (CP) or in adults with hemiplegia [24,25,26,27,28,29,30]. We have tried to compare some of the results, while keeping cautions.

The most important correlation we have found was the dependence between asymmetrical tonic neck reflex and pelvic kinematics. We have found that pelvic obliquity symmetry depended on levels of ATNR activity. The children with a higher index of ATNR had a lower index of symmetry pelvic obliquity. There was a significant correlation for all of the tests done. Both the standing test and the quadruple test results correlated with symmetry pelvic obliquity. This means that checking ATNR in open- and in closed-chain gives comparable results. Moreover, both the right and the left form of ATNR have an impact on symmetry in the frontal plane of the pelvic kinematics during gait. We have observed similar results for pelvic rotation symmetry. All results have a negative correlation to ATNR level. The results for the right side of the ATNR were significant. We have found no correlation of ATNR level with pelvic tilt. The explanation for these results can be based on the mechanical pattern of the reflex. To describe it closer, we analyzed the movement of the body during the reflex response in parts. During the head’s turn to the right, the right leg and arm moved to extension and at the same time the left arm and leg moved to flexion. This kind of movement or muscle tenseness must impact pelvic motion. Pelvic motion can be visible in various forms. A high level of reflex activity gives a more obvious response in the form of body movement, especially in the limbs. A low level of reflex activity leads to muscle tension, sometimes even invisible. The change in tension in some parts of the body, in the example stated above, may determine the pelvic kinematics in terms of both the obliquity and rotation. The pelvic kinematics are changed by additional movements of the limbs and trunk in the response of the reflex reaction. This is obviously a model of pelvic behavior as an effect in the case of persistent reflex. We know that more specific movement analysis is needed. Our observation is described first and should lead to deeper analysis with different research devices. We can see the need for wider analysis of the body’s reaction in the ATNR response in spontaneous child movement as well as movement with a given task. For such observation, a tool for in-depth motion analysis is necessary.

Pelvis symmetry index in children is not evaluated as often. In the literature, we can find absolute symmetry index (ASI) calculated, which is based commonly on foot loading [31]. As this is a different method of checking symmetry in gait, it does not directly indicate pelvis symmetry. Asymmetry in pelvic obliquity is very common in spastic cerebral palsy and scoliosis [24,32]. It is a parameter observed in static position. The results are obvious, taking into consideration the severity of the illnesses. In our study, children were symmetrical in clinical images but the asymmetries became visible during movement. Manicolo et al. and Papadopoulos et al. conducted analyses of gait symmetries in children with ADHD and developmental coordination disorders [31,33,34,35,36,37]. The authors show many gait variables in these groups. Some of the spatiotemporal gait variables in ADHD children were not different than in the control group [34]. These results show the disturbances in gait in such illnesses.

Performing further analyses in our research, we also divided children into two sub-groups by age. The only statistically important difference we found between these groups was pelvic obliquity (F). The symmetry in this parameter was higher in the younger group (under 5 years old) than in the group above 5 years old. Smith et al. [38] also checked the gait parameters of normally developed children. This work was conducted in South Africa. The children’s range of pelvis tilt was around 18 degrees, and pelvis obliquity was around 6 degrees as well as pelvis rotation. In our group, we observed a range of motion for pelvic tilt around 2.8 degrees, for pelvic obliquity 5.8 degrees, and for pelvis rotation around 15 degrees. A very big difference in the results was found in pelvic tilt and pelvic rotation. Can it be the result of ATNR activity? It is not so obvious. The differences may be rather enlarged by the different races of examined children and are very intriguing for future investigation. The authors, like us, compared gait parameters in two groups of children, in this case aged 6–8 years old and 9–10 years old. Smith at al.’s research shows minimum variation in the kinematic patterns of the pelvis between the two age groups. There was no statistical difference in the results between the two groups. As the authors took into consideration older children than in our research, the comparison cannot be literal and this may be the next reason for such discrepancies.

In the sub-groups dependent on gender, we found a statistical difference in pelvic tilt (S) symmetry. Boys obtain a significantly higher result for this parameter than tested girls. The differentiation in pelvic tilt symmetry between genders may also be connected with a difference in sagittal standing posture between genders, such as pelvic tilt which is higher in girls than in boys [39]. The higher range of motion makes the system more unstable, thus the results showing higher asymmetrical pelvic tilt in girls is the consequence.

As there is little research connected with the gait symmetry index in normally developing children, a wider comparison is not possible. Most of the studies focus on spatio-temporal gait parameters, which is not the field of our study. Even if kinematic parameters are explored in some studies, the symmetry index of pelvis motion has not been found by authors so far. The impact of primitive reflexes on a child’s life is still being checked by different kinds of researchers in different disciplines such as psychology, pedagogy, and physiotherapy, where some interventions are being undertaken [38,39,40,41,42,43]. This demonstrates the importance of cooperation for child integral understanding and support. The differentiation in gait patterns completes the child picture with primitive reflexes in clinical images. This kind of child movement is usually described as clumsy. Our study may introduce a reason for a specific way of moving. Movement in which the child cannot control the movement of his or her limbs causes many problems. The child’s physically awkward behavior can be incomprehensible for his or her entourage. Such analysis as that which we have conducted may explain the child’s unwitting behavior as well as remind and emphasize how important it is to work on the integration of primitive reflexes for a well-balanced child.

## 5. Conclusions

This research can bring important information for physicians, physiotherapists, and parents taking care of children with certain developmental disturbances. The awareness of active primitive reflexes contributing to misbalancing so many spheres of child development may be essential in the decision-making process regarding the facilitation, management, modification, or elimination of each sign of developmental delays.

## Figures and Tables

**Table 1:** Range of motion, symmetry index and descriptive statistics of pelvic motion.

Pelvic Tilt (S)							
	L Side			R Side			
	Back Tilt	Front Tilt	Range	Back Tilt	Front Tilt	Range	Symmetry Index
MEAN	1.8	0.9	2.8	1.9	0.9	2.8	55.2
MAX	3.3	3.3	5.1	3.3	3.8	6.5	99.3
MIN	0.6	0.1	1.1	0.4	0	0.7	5.2
SD	0.7	0.7	1.1	0.7	0.7	1.2	25.3
**Pelvic Obliquity (F)**							
	**L Side**			**R Side**			
	**Low**	**Up**	**Range**	**Low**	**Up**	**Range**	**Symmetry Index**
MEAN	2.9	2.9	5.8	2.8	2.9	5.8	95.1
MAX	9	8.3	17.3	7.8	9.2	17	99.5
MIN	0.7	0.6	1.6	0.7	0.4	1.7	55.4
SD	1.6	1.5	3.0	1.4	1.7	3.0	8.4
**Pelvic Rotation (T)**							
	**L Side**			**R Side**			
	**External**	**Internal**	**Range**	**External**	**Internal**	**Range**	**Symmetry Index**
MEAN	7.7	7.7	15.4	7.1	8.0	14.8	93.2
MAX	14.7	16.7	31.4	14.8	14.1	26.1	99.5
MIN	1.9	2.6	5.6	1.5	1.8	3.4	40.3
SD	2.7	3.0	5.3	3.2	2.9	6.1	10.5

**Table 2:** Spearman correlation between asymmetrical tonic neck reflex and gait symmetry index.

Gait Parameters	Gait Symmetry Index		
	Pelvic Tilt (S)	Pelvic Obliquity (F)	Pelvic Rotation (T)
ATNR R	0.048	−0.407 **	−0.307 *
ATNR L	0.056	−0.430 **	−0.249
ATNR in standing R	0.053	−0.387 **	−0.384 **
ATNR in standing L	0.041	−0.341 *	−0.260

* *p* < 0.05; ** *p* < 0.01.

**Table 3:** Gait parameters comparison between the two groups of younger and older children.

Symmetry	Under 5 Years Old			Above 5 Years Old			U	*p*	η^2^
	M	Me	SD	M	Me	SD			
Pelvic tilt (S)	56.75	56.80	22.72	51.53	59.20	27.25	267.50	0.528	0.01
Pelvic obliquity (F)	97.82	98.70	2.16	94.18	97.60	9.24	178.00	0.015 *	0.12
Pelvic rotation (T)	94.60	97.80	8.03	92.83	97.30	12.17	272.50	0.595	0.01

M—mean; Me—median; SD—standard deviation; U—Mann–Whitney test results; *p*—level of significance; η^2^—effect size; * *p* < 0.05.

**Table 4:** Gait symmetry parameters due to gender of examined children.

Gait Parameters	Girls (*n* = 30)			Boys (*n* = 20)			*U*	*p*	η^2^
	M	Me	SD	M	Me	SD			
Pelvic tilt (S)	47.15	37.70	27.49	63.89	69.30	17.42	181.50	0.027 *	0.10
Pelvic obliquity (F)	97.17	98.50	3.53	94.03	97.60	10.09	201.50	0.072	0.06
Pelvic rotation (T)	92.13	97.30	12.88	95.87	97.60	4.29	286.00	0.935	0.00

M—mean; Me—median; SD—standard deviation; U—Mann–Whitney test results; *p*—level of significance; η^2^—effect size; * *p* < 0.05.
